# Can cactus (Opuntia stricta [Haw.] Haw) cladodes plus urea replace wheat bran in steers’ diet?

**DOI:** 10.5713/ajas.17.0927

**Published:** 2018-05-24

**Authors:** Maria Gabriela da Conceição, Marcelo de Andrade Ferreira, Janaina de Lima Silva, Cléber Thiago Ferreira Costa, Juana Catarina Cariri Chagas, Carolina Corrêa de Figueiredo Monteiro

**Affiliations:** 1 Depatment of Animal Science, Federal Rural University of Pernambuco, Recife, PE 52171-900, Brazil; 2 Multidiciplinary Center of Barra, Federal University of West Bahia, Barra, BA 47100-000, Brazil; 3 Department of Agriculture, Sertão of Pernambuco Federal Institute, Floresta, PE 56316-686, Brazil; 4 II Campus, Federal University of Alagoas, Santana do Ipanema, AL 57.500-000, Brazil

**Keywords:** Degradable Ruminal Nitrogen, Microbial Protein Synthesis, Ruminal Fermentation, Cactus Cladodes

## Abstract

**Objective:**

The study aimed to evaluate the effect of replacing wheat bran for cactus cladodes plus urea (0%, 25%, 50%, 75%, and 100%) on the intake of nutrients, nitrogen balance, microbial protein synthesis, and rumen fermentation for steers.

**Methods:**

Five crossbred steers (1/2 Holstein-Zebu), with rumen cannula and an average body weight of 180±5.3 kg, were assigned to a 5×5 Latin square design. Dietary treatments consisted of the replacement of the total of wheat bran in basal diet by cactus cladodes using the following proportions: 0% for basal diet, 25%, 50%, 75%, and 100% cactus cladodes replacing wheat bran. Urea was added to the diets to adjust the crude protein (CP) content to 130 g/kg dry matter.

**Results:**

Maximum dry matter intake (5.73 kg/d) and maximum nitrogen balance (103 g/d) were estimated for 54.6% and 70.8% replacement levels of wheat bran. The maximum microbial protein production (44.6 g/d) was obtained at a replacement level of 49.7%, and a medium value (125 g CP mic/kg total digestible nutrients) of microbial protein efficiency was observed. The rumen pH increased linearly according to cactus cladodes inclusion, while the ammonia nitrogen medium value was 24.5 mg/dL.

**Conclusion:**

The replacement of 55% wheat bran for cactus cladodes plus urea in the diet of crossbred steers is recommended.

## INTRODUCTION

In semiarid regions, the occurrence of frequent droughts determines the success of economic activities such as livestock and agriculture, due to the seasonality of forage production. To overcome this problem, farmers are forced to import grains from other regions to meeting the animals’ nutrient requirements, representing an increase in production cost. A greater feed ingredient in ruminant nutrition is the wheat bran, a byproduct with a high energy content and relevant crude protein (CP) [1], however its inclusion in diet represents increases in the cost, once US$ 0.22 per kg of dry matter (DM).

In this scenario, the use of local alternative feeds as cactus cladodes encourages the livestock sector [2] been an essential to sustainability of the productive system in arid and semiarid regions [3]. The cactus cladodes present adaptive agronomic characteristics, such as being tolerant to hydric stress, and being suitable as forage in diets for dairy goats, dairy cattle, and sheep [4–6], respectively. It also has a high content of non-fibrous carbohydrates (NFC), and consequently significantly high energy content compared with other forage plants [7,8]. Nonetheless, the cactus cladodes are poor in protein, and thus the non-protein nitrogen (NPN) compounds, such as urea, should be used to increase dietary protein content, due to its low cost compared to true protein sources such as soybean meal [9].

The urea can totally replace the protein ingredient in bovine diets confined fed medium concentrated meal for 1 kg/d of average daily gain [10]. The elevations in the hepatic ureagenesis could increase the losses of urine nitrogen, compromising the utilization efficiency of the dietary nitrogen compounds [11]. It has long been recognized that supplemental NPN is most efficiently utilized in rations low in protein and relatively high in digestible energy. However, the maximum level of inclusion of urea in ruminant diet that compromising the performance it not clear.

In these assumptions, it was speculated that due to the high content of carbohydrates rapidly fermentation in the rumen in diets based on cactus cladodes, it was possible to raise the level of urea without compromising animal intake and metabolic condition, since a greater amount of ammonia could be assimilated by the rumen microbiota. Thus, this study aimed to evaluate the effect of replacing wheat bran for cactus cladodes plus urea on the N balance, microbial protein synthesis, and rumen fermentation in crossbred steers.

## MATERIALS AND METHODS

### Animal care

The management and care of animals were performed in accordance with the guidelines and recommendations of the Committee of Ethics on Animal Studies at the Federal Rural University of Pernambuco (License N°009/2015), Recife, Brazil. This study was carried out in the Department of Animal Science at the Federal Rural University of Pernambuco, located in Recife, Pernambuco State, Brazil.

### Animals and diests

Five rumen fistulated steers (1/2 Holstein-Zebu) with an average initial body weight (BW) of 160±5.3 kg were assigned in a 5×5 Latin square design. The experiment lasted 80 days, corresponding to five 16-day periods. The first seven days were allocated to the adaptation of the animals to the experimental diets according to [12,13], followed by nine days of sample collections. The animals were weighed, identified and vermifuged prior to the experiment and housed in individual pens equipped with feeders and drinkers.

### Experimental procedure and chemical

The chemical composition of the ingredients and proportion of the ingredients in the concentrate mixture and the chemical composition of the diets are shown in Table 1 and 2. Dietary treatments consisted of the replacement of the total of wheat bran in basal diet by cactus cladodes using the following proportions: 0% for basal diet, 25%, 50%, 75%, and 100% cactus cladodes replacing wheat bran. Urea was added to the diets to adjust the CP content to 130 g/kg DM.

The fresh sugar cane and cactus cladodes were cut and chopped daily and then provided to animals. The DM content of sugarcane and cactus cladodes was evaluated weekly to adjust the amount of feed allowed to the animals. The mixture of ingredients was performed manually in the feeders, highlighting that the cactus cladodes mucilage allowed a uniform aggregation of urea. The diets were supplied *ad libitum*, allowing approximately 100 g/kg as orts. The animals were fed twice daily in equal portions at 06.00 h and 18.00 h. Water was provided *ad libitum* throughout the experimental period.

The intake of DM and nutrients from the diets was calculated as the difference between the total nutrients in the feed offered and the total nutrients present in the orts. Forage provided and orts were sampled daily during the collection period and subjected to partial drying in a forced ventilation oven set at 60°C for 72 h. The ingredients that comprised the concentrate were sampled directly from the feed mill silos on the days that they were mixed. All samples were processed in a Wiley mill to pass through a 2-mm screen sieve. After that each sample was homogenized and divided in two portions. Half of each sample was processed again in the same mill to pass through a 1-mm screen sieve.

The samples processed to pass through the 1-mm screen sieve were evaluated for DM (method INCT-CA G-003/1), organic matter (OM; method INCT-CA M-001/1), CP (method INCTCA N-001/1), ether extract (EE; method INCT-CA G-005/1), neutral detergent fiber corrected for ash and protein (NDFap; methods INCT-CA F-002/1, INCT-CA M-002/1, and INCT-CA N-004/1), and neutral detergent insoluble protein (method INCT-CA N-004/1), according to the standard techniques of Brazilian National Institute of Science and Technology in Animal Science (INCT-CA) [14]. Feeds and orts samples processed to pass through the 2-mm screen sieve were evaluated for the indigestible NDF (iNDF; method INCT-CA 009/1) contents by using a 288 hours *in situ* incubation procedure.

The NFC contents were quantified according to [15] as follows: NFC = OM–[(CP–CPu+U)+NDF+EE +MM]; where CPu = CP content from urea, U = urea content, and NDF = NDFap corrected for residual ash and protein. The other terms were previously defined and all of them are expressed as g/kg DM.

The total digestible nutrients (TDN) were determined according to [16]: TDN = CP_d_+NFC_d_+NDF_d_+EE_d_×2.25 (subscript means digestible).

For three days in each experimental period, after providing the morning diet, total feces and urine collection (24 h) were performed, and the pH (the urine) was measured every 6 h to make sure that it is below 3.0. To collect urine samples, funnel collectors were coupled to animals and attached to hoses used to conduct urine to a container containing 500 mL of 20% sulfuric acid. At the end of each collection period, the weight and total volume of urine were measured, and the total N content was determined using the Kjeldahl method INCT-CA N-004/1 [14]. The urinary purine derivatives (PD) were quantified using the colorimetric [17]. Urea (blood and urine) levels were measured from commercial kits, using a colorimetric system in a semi-automatic biochemical analyzer D-250 (Labtest, Lagoa Santa, MG, Brazil). Blood samples were collected after 11 days in each experimental period, 4 h after the morning feeding, through a puncture in the jugular vein using vacutainer tubes containing a separation gel with a coagulant activator (SST II Advance, BD Vacutainer, Curitiba, PR, Brazil). The samples were immediately centrifuged (5,000 rpm for 20 min) to remove plasma and analyze the urea content.

The N balance estimate was obtained by subtracting the fecal and urinary excretion values from ingested N. To determine the efficiency of dietary N compound utilization, the following indicators were used: N-urea in plasma, urinary excretion of N-urea, and N balance. The urea-N from plasma and urine was estimated using the factor 0.466 [18].

The total excretion of PD was calculated as the sum of the quantities of allantoin and uric acid excreted in the urine. Absorbed purine concentrations (X, mmol/d) were calculated using the equation Y = 0.85X+0.385BW^0.75^ [19].

The rumen synthesis of N compounds (Nmic, g N/d) were calculated using the equation Nmic = (70×AP)/(0.83×0.116× 1,000) [17].

Rumen fluid was collected from the 11th to 13th day of each experimental period, before feeding and two, four, and six hours after feeding. The manual collection was performed at several locations in the ruminal environment, taking a representative sample of the content (100 mL), which was filtered through cotton fabric. After collection, pH was measured with a digital potentiometer. Immediately after collection, the liquid was frozen (−15°C) for later analyses. The ammonia nitrogen (N-NH_3_) concentration was determined after samples were centrifuged at 3,000 rpm/15 min, using the supernatant for analysis by Kjeldahl method INCT-CA N-007/1 [14].

### Statistical analyses

The variables studied were analyzed with the PROC MIXED option in SAS software (version 9.4), adopting 0.05 as the critical level of probability for type I error, according to the following model:

$${\text{Y}}_{\text{ijk}}=\mu +T_{\text{i}}+a_{\text{j}}+p_{\text{k}}+{ɛ}_{\text{ijk}}$$

Where, Yijk is the dependent variable measured in animal j that was subject to the i treatment in period k; μ is the general mean; *T*_i_ is the fixed effect of treatment i; *a*_j_ is the random effect of animal j; *p*_k_ is the random effect of period k; and ɛ_ijk_ is the unobserved random error assuming normal.

After analysis of variance, the significance of the linear and quadratic effects of the replacement of the total of wheat bran in the basal diet by cactus cladodes was evaluated. Rumen pH and NH_3_-N were considered the effect of sampling time as repeated measures in time.

## RESULTS

Intake of DM, CP, and nitrogen as excretion of urinary nitrogen and nitrogen balance demonstrated a quadratic pattern, whereas excretion of fecal nitrogen decreased linearly (p<0.01; Table 3). The maximum values of intake of DM (5.73 kg/d), CP (830 g/d), N (132 g/d), N balance (103 g/d) and minimum value of urinary nitrogen (11.5 g/d) were estimated at 54.6%, 56.7%, 56.8%, 70.8%, and 76.7% of replacement of wheat bran for cactus cladodes plus urea, respectively.

The plasmatic concentration of urea and urea-N presented a quadratic pattern (p<0.01; Table 4), whereas the maximum plasma levels of urea (41.9 mg/dL) and urea-N (19.5 mg/dL) were estimated for 55.1% and 54.9% of replacement of wheat bran for cactus cladodes plus urea, respectively. The urea excretion in urine increased linearly by 3.05 mg/kg BW for every 1% increase in replacement levels (Table 4).

The microbial-N and microbial protein presented a quadratic response in accordance with the replacements levels (p<0.01; Table 4). The maximum microbial-N (49.9 g/d), microbial protein (443.9 g/d) were estimated at 74.0, 49.7 of replacement of wheat bran for cactus cladodes plus urea, respectively. The microbial protein efficiency (125.2 g CP mic/kg TDN) was not altered by the replacement (p>0.05; Table 4).

**Figure 1:**
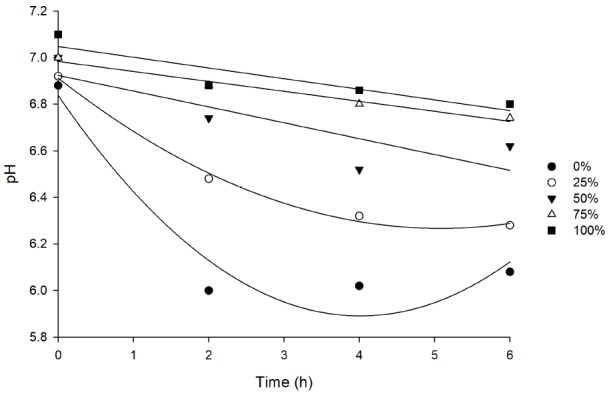
pH values for different replacement levels (0%, 25%, 50%, 65%, 100%) of wheat bran for cactus cladodes plus urea through the time.

The replacement levels influence the pH which increased according to cactus cladodes plus urea increment (p<0.001; Table 5). There was an interaction between time and replacement for pH, and a deployment was made (p<0.01; Table 5). For the replacements levels of 0% and 25% were observed as a quadratic pattern through the time, where the minimum pH values were 5.92, 6.05 at 3.96, and 5.92 hours, respectively, after feeding (Figure 1). Instead of it, for all collection time after feeding, the pH increased linearly according to cactus cladodes plus urea inclusion in the diets (Table 6).

The rumen NH_3_-N varied through time, presenting a quadratic pattern with a maximum value of 31.9 mg/dL estimated at 3.82 h after feeding. For the different replacement levels of wheat for cactus cladodes, the mean value for NH_3_-N was 24.52 mg/dL (Table 5).

## DISCUSSION

Replacement of 54.6% wheat bran with cactus cladodes allowed a greater nutrient intake, which suggests that cactus cladodes, owing to its chemical characteristics (Table 1), improved the flow of digestion through the gastrointestinal tract, resulting in increased intake [20]. The negative effect of greater levels of cactus cladodes in diets on voluntary intake could be associated with the high moisture content in this feed, which increases its capacity to occupy space in the rumen environment [21]. According to [22], water content in forages exceeding 700 g/kg can compromise voluntary intake. Moisture of the greater level of cactus cladodes diet was estimated at 840.3 g/kg.

In parallel to the above, it should be considered the animals’ urea intake amount calculated at 83 g/100 kg BW for 54.6% of replacement level is considered high up in the recommendations for ruminants (0.45 to 0.50 g/kg BW) [23]. High level of urea in diet decreases the palatability and rises the intoxication risk due to its greater solubility in the rumen and consequent absorption [24]. Concomitantly, the increases in ruminal pH facilitates the rapid transport of ammonia across the rumen epithelium, resulting in a rapid increase in blood ammonia [25]. In accordance to the exposed in Table 5, a linear increase was noted due to the replacement, insofar there was an increase in plasma urea concentration and urea excretion in urine (Table 4), confirming that those aspects may contributed to the DM intake drop for the replacement level from 54.6%.

The maximum recommendation of urea seems to be somewhat conservative, since, despite being associated to DM intake depression, the animals did not show symptoms of intoxication over the experimental period, even considering that the urea intake was twice of the amount recommended by the cited authors. Thus, the observed values for urea inclusion in the diets ratify the recommendation of [10] who suggested that urea can totally replace the protein ingredients in bovines’ diets, guaranteeing moderated daily gains (0.8 to 1.0 kg/d).

At replacement levels above 54.6%, the input of N rumen degradable (urea) increased significantly, promoting an excess, which is also evidenced by the increase of plasma urea-N (Table 4). According to [26], concentrations between 14 and 16 mg/dL of plasma urea-N in Zebu steers allows for maximal microbial efficiency, and above these levels, as observed in this study (19.5 mg/dL for 54.9% replacement; Table 3), dietary protein losses begin [27]. The excess urea concentrations, as demonstrated by increased excretion in the urine [28] and plasma (Table 3), indicate the impairment of diet palatability (bitter flavor of urea) and rumen fermentation. In this case, it may be inferred that the energy released by NFC fermentation was not sufficient to assimilate the large amount of readily available N (NPN) from rumen microorganisms.

The reduction in the loss of N in the feces (Table 3) can likely be explained by the increased use of NH_3_-N by rumen microorganisms (Table 5). Furthermore, the smaller N loss in the urine (estimated at 76.5% replacement of wheat bran) suggests the largest conversion of N to urea, which is seen in the increased urinary excretion of urea (Table 3).

According to [29], when the rumen protein degradation rate exceeds the rate of carbohydrate degradation, an increase in the excretion of nitrogenous compounds and urea production occurs. Due to the higher energy costs for urea synthesis in animals [30], a reduction of the energy available for microbial protein synthesis occurs, confirming the results obtained for this variable above the 50 % replacement level of wheat bran (Table 4).

The maximum N balance (NB) estimated at a 70.8% replacement level of wheat bran with cactus cladodes (Table 3) complements the results obtained for N excretion in urine and feces in that the lower excretion of this nutrient indicates higher retention in the organism. The protein utilization in animal metabolism at replacement levels close to 75% of the DM of diets justifies microbial efficiency pattern and no replacement influence (medium value of 125.2 g microbial CP/kg TDN; Table 4), consistent with results proposed by [31] for Zebu crosses where 120 g microbial CP/kg TDN was used as a reference for microbial efficiency in tropical conditions.

There was a linear increase in rumen pH, demonstrating an improvement in the rumen environment equilibrium with the cactus cladodes plus urea inclusion (Table 5). The mucilage in cactus cladodes may stimulate salivation, thus preventing pH decrease [2]. Also, the greater pH values may be associated with the biggest amount of urea input in rumen according to increased replacement levels, as mentioned above, due to its rapid solubilization with consequent ammonia amassing, which has a well-known alkaline potential [32,33].

To highlight the difference in carbohydrate profiles for the diets, it is seen that those with more inclusion of wheat bran present a more content of starch versus cactus cladodes diets, which directly impacts the rumen fermentation pattern and is confirmed in Figure 1. The diet with no cactus cladodes inclusion shows a rumen pH minimum of 5.92 estimated at 3.96 hours after feeding vs a linear decreasing (7.10 to 6.8; Table 6) in ruminal pH for diets with 50.51% of cactus inclusion.

The concentration of NH_3_-N in the rumen is important for microbial growth, and is largely dependent on the amount of substrate and the fermentation of the OM present in the rumen. In this study, an increase in the intake of NPN, derived from urea, probably resulted in a higher concentration of rumen NH_3_-N (24.5 mg/dL), as found by [34]. The maximum rumen NH_3_-N presented is accordance with the amount (10 to 23 mg/dL) suggested by [35] for maximum for maximum rumen fermentation activity and microbial growth.

When urea is supplied in the diet of ruminants, the peak rumen ammonia is usually 1 or 2 h after a meal, as results proposed by [36]. In this study, the maximum concentration of rumen NH_3_-N (31.9 mg/dL) occurred only 3.82 h after feeding. This result may be related to the quality of the fiber used in the diets, derived from sugar cane and wheat bran, where the association made the microbial fermentation slower, thus promoting greater retention of food and delaying the peak in NH_3_-N.

On the basis of the results of this study, replacement of 55% wheat bran with cactus cladodes in the diet of steers is recommended to promote a better DM intake and microbial protein synthesis. In semi-arid regions, the cactus cladodes can be considered as an alternative to wheat bran without altering microbial synthesis efficiency, which contributes to animal production in periods of forage restriction.

## IMPLICATIONS

The paper provides brand new about animal production and nutrition information, relating to cactus cladodes plus urea inclusion in cattle diets. Due to semiarid climate condition, there is no expressive grains production, raising animal’s feeds cost. Thus, the information presenting proposes the use maximization of cactus cladodes plus urea in replacement to wheat bran, which guarantees lower prices to feed the cattle and the system production sustainability in semiarid areas.

## Figures and Tables

**Table 1:** Chemical composition of ingredients (g/kg of dry matter)

Item	Ingredients (g/kg)			

	Cactus cladodes	Sugar cane	Wheat bran	Corn
Dry matter, as fed	105.5	282.5	867.3	864.8
Organic matter	802.5	980.2	949.0	986.2
Crude protein	55.5	18.7	152.4	77.0
Ether extract	12.1	11.2	29.8	39.3
Neutral detergent fiber1)	291.6	410.1	326.6	107.9
Indigestible neutral detergent fiber	118.3	239.3	101.7	12.7
Non fiber carbohydrates	406.7	488.4	399.7	748.4

1) Corrected to ash and protein.

**Table 2:** Ingredients proportions and chemical composition of dietary treatments for steers fed cactus cladodes plus urea

Item	Replacement levels (%)				

	0	25	50	75	100
Ingredients (g/kg DM)					
Sugarcane	376.3	369.9	367.6	362.1	371.4
Cactus cladodes	0.0	129.6	259.2	388.8	505.1
Wheat bran	518.4	388.8	259.2	129.6	0.0
Corn	77.0	78.5	75.9	76.6	75.6
Urea:ammonium sulfate (9:1)	14.3	19.2	24.1	28.9	33.9
Minerals	14.0	14.0	14.0	14.0	14.0
Chemical composition (g/kg DM)					
Dry matter, g/kg as fed	488.4	320.9	238.4	189.9	159.7
Organic matter	937.0	913.3	889.5	865.9	844.4
Crude protein	129.9	130.3	135.5	130.6	130.6
Ether extract	22.9	20.4	17.9	15.6	13.2
Neutral detergent fiber1)	331.9	324.1	319.2	312.2	307.7
Indigestible neutral detergent fiber	143.7	144.4	145.9	146.8	149.6
Non-fibrous carbohydrates	448.6	447.5	445.4	444.1	443.4
Total digestible nutrients2)	683.6	708.9	673.8	642.6	628.4

DM, dry matter.

1) Correction to ash and protein.

2) Estimated by the equation [37].

**Table 3:** Nitrogen intake, excretion and balance for steers fed cactus cladodes plus urea

Item	Replacement levels (%)					SEM	p value1)	
	
	0	25	50	75	100		L	Q
Daily intake								
Intake of dry matter (kg/d)	4.08	5.47	5.88	5.12	4.84	0.392	0.146	<0.001
Intake of crude protein (g/d)	515	737	821	765	729	53.84	<0.001	0.001
Nitrogen intake (g/d)	82.3	118	131	122	117	8.613	<0.001	0.001
Nitrogen excretion (g/d)								
Fecal nitrogen	28.1	22.4	20.9	18.2	15.6	1.422	<0.001	0.219
Urinary nitrogen	28.8	21.0	11.0	13.5	12.6	3.371	0.049	0.019
Nitrogen balance	25.4	74.6	99.4	90.7	88.5	9.210	<0.001	<0.001

SEM, standard error of the mean; L, linear effect; Q, quadratic effect.

1) Cubic effect was not significant.

**Table 4:** Plasmatic concentration, excretion of urea and N-ureic, and microbial protein synthesis for steers fed cactus cladodes plus urea

Item	Replacement levels (%)					SEM	p value1)	
	
	0	25	50	75	100		L	Q
Plasma (mg/dL)								
Urea	32.3	41.2	42.3	38.3	37.3	2.92	0.368	0.018
Urea-N	15.0	19.2	19.7	17.9	17.4	1.36	0.369	0.018
Urine (mg/kg BW)								
Urea	642	803	686	1,019	915.7	48.15	<0.001	0.367
Urea-N	379	374	360	431	432	42.24	<0.001	0.367
Microbial protein								
Microbial protein synthesis (g/d)	355	426	451	409	361	13.80	0.809	<0.001
Microbial protein efficiency (g CP mic/kg TDN)	133	120	116	133	124	10.26	0.966	0.240

SEM, standard error of the mean; L, linear; Q, quadratic. BW, body weight; CP, crude protein; TDN, total digestible nutrient.

1) Cubic effect was not significant.

**Table 5:** Ruminal fermentation for steers fed cactus cladodes plus urea

Item	Replacement levels (%)					SEM	p value		
	
	0	25	50	75	100		Treat	Time	Treat×time
pH	6.24	6.50	6.72	6.85	6.91	0.07	<0.001	<0.001	0.015
Ammonia-N (mg/dL)	25.0	29.1	26.5	21.8	20.2	4.21	0.561	<0.001	0.455

SEM, standard error of the mean; Treat, treatment (replacement levels).

**Table 6:** Average means of ruminal pH in function of collection times for steers fed cactus cladodes plus urea

Replacement level (%)	Collection times (h)				p value1)	
	
	0	2	4	6	L	Q
0	6.88	6.00	6.02	6.08	0.004	0.008
25	6.92	6.48	6.32	6.28	<0.001	0.013
50	7.00	6.74	6.52	6.62	0.010	0.096
75	7.00	6.88	6.80	6.74	0.009	0.639
100	7.10	6.88	6.86	6.80	0.003	0.166
L	0.167	<0.001	<0.001	<0.001	-	-
Q	0.926	0.154	0.167	0.170	-	-

L, linear effect; Q, quadratic effect.

1) Cubic effect was not significant.
