# Corrigendum

**DOI:** 10.5713/ajas.17.0927C

**Published:** 2018-10-23

The following is the updated title of **Asian-Australas J Anim Sci 2018. Vol 31, No. 10 : 1627–1634**.

Can cactus (*Nopalea cochenillifera* Salm Dyck) cladodes plus urea replace wheat bran in steers’ diet?

